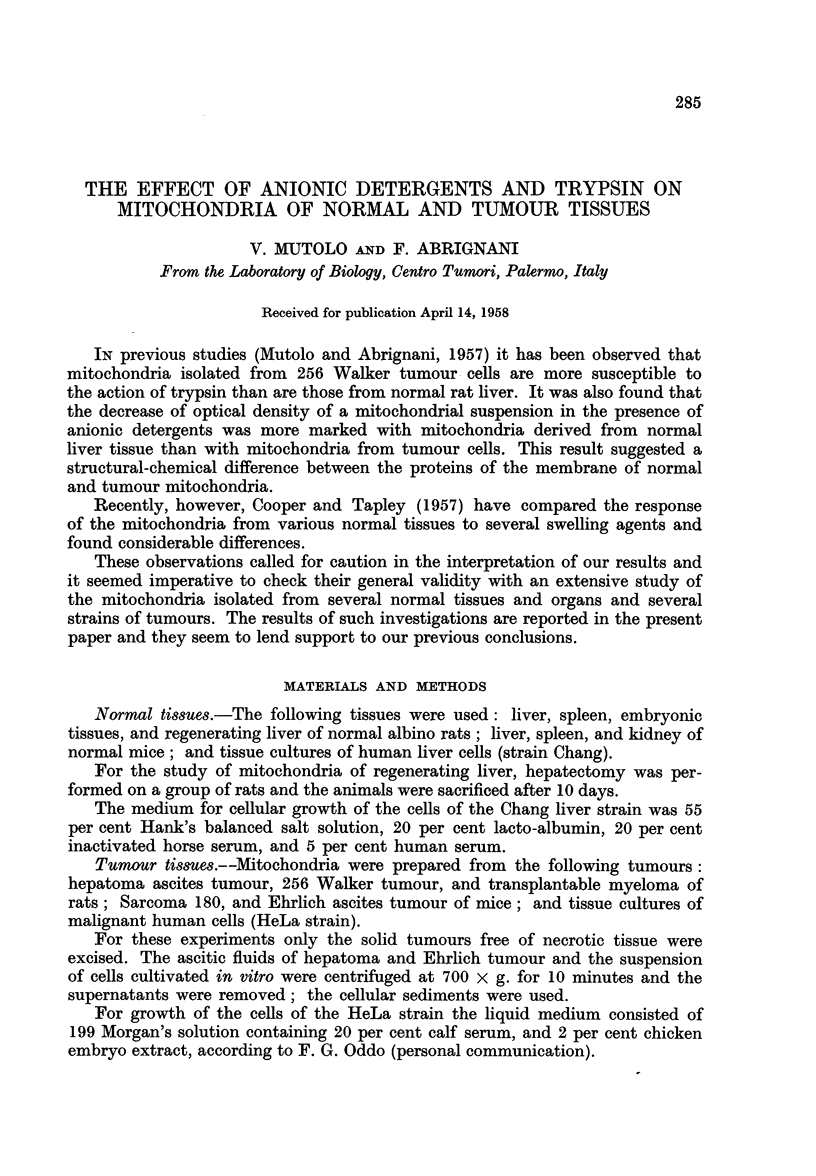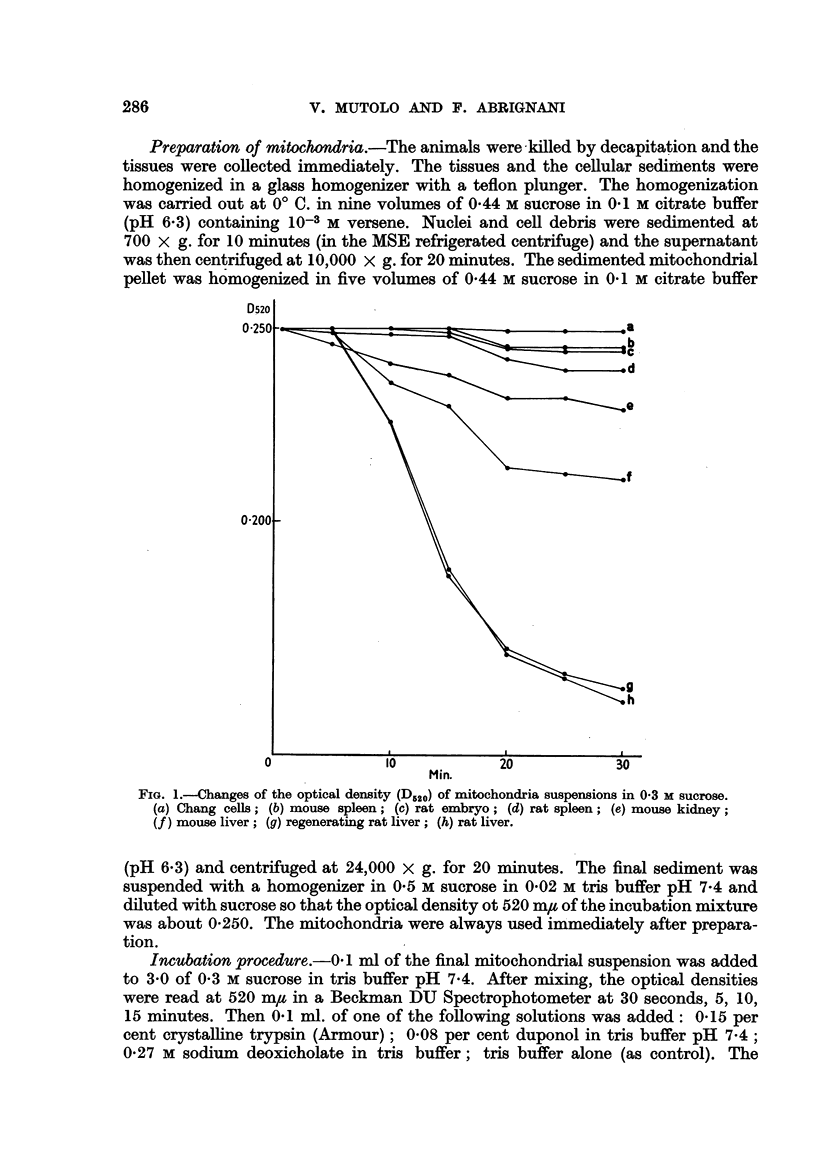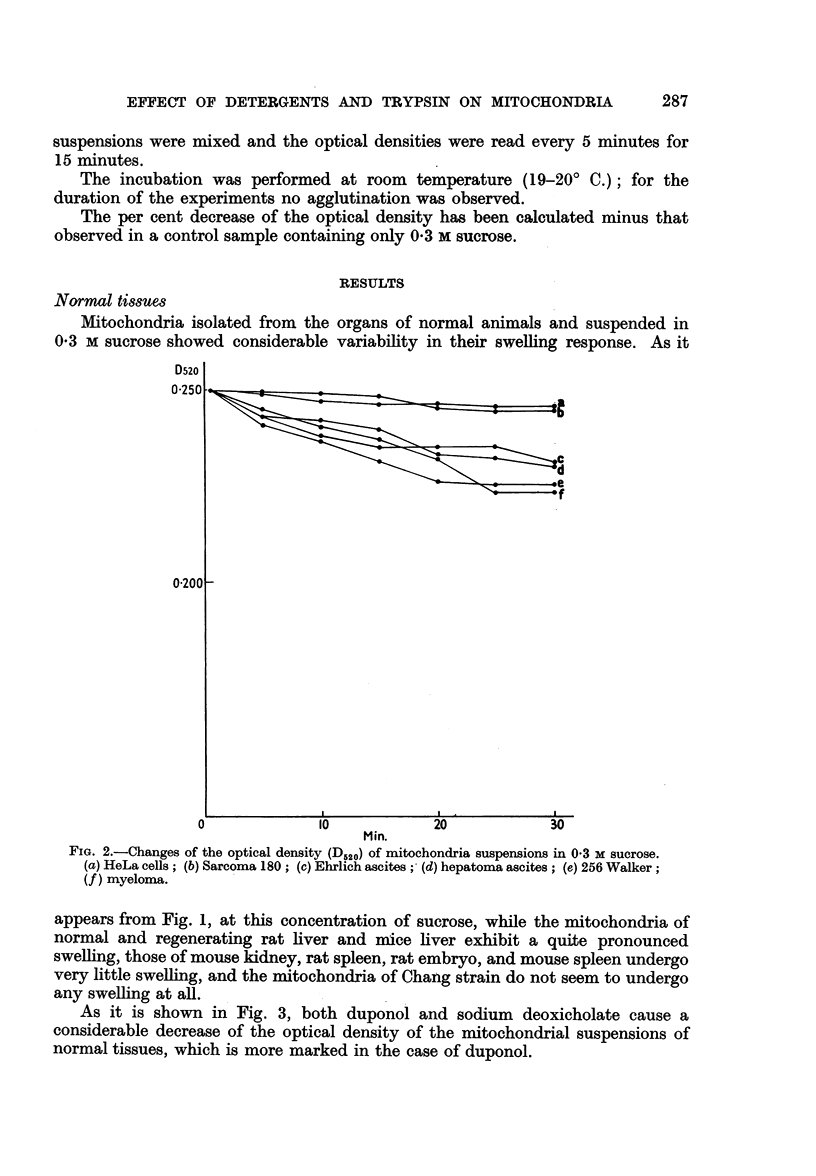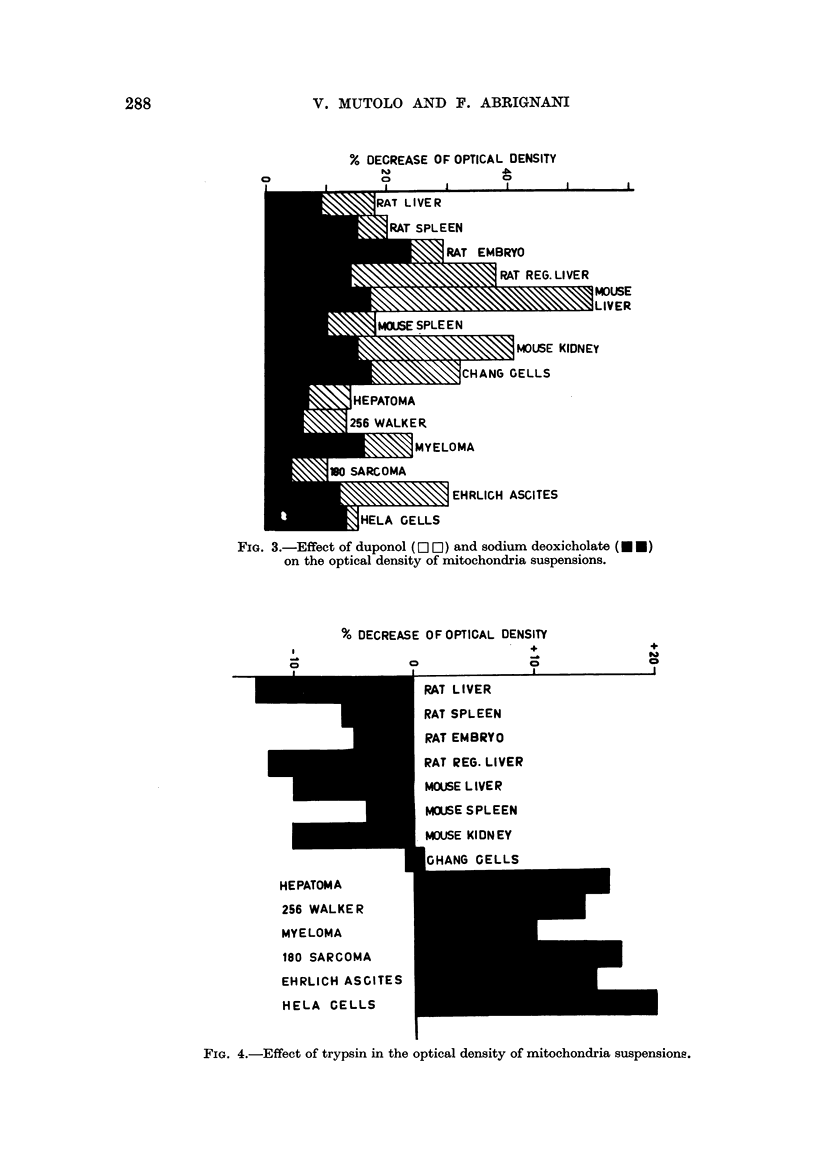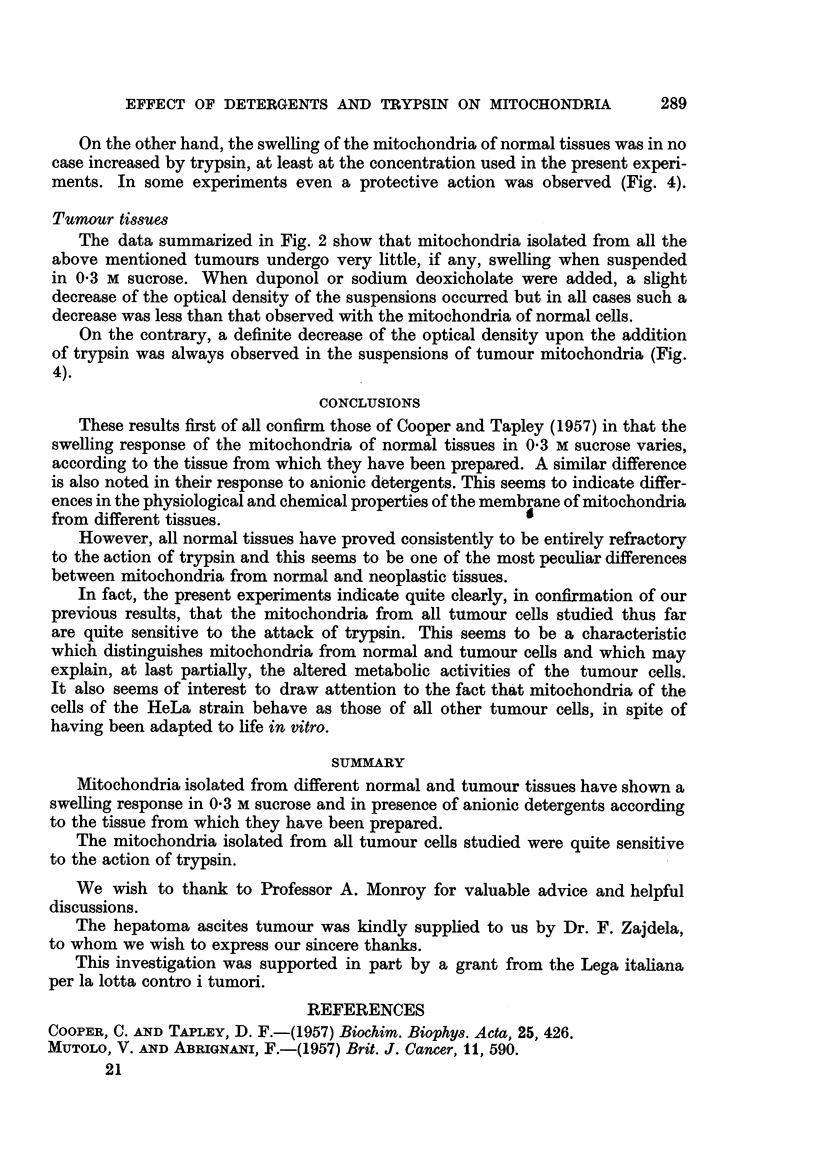# The Effect of Anionic Detergents and Trypsin on Mitochondria of Normal and Tumour Tissues

**DOI:** 10.1038/bjc.1958.34

**Published:** 1958-06

**Authors:** V. Mutolo, F. Abrignani


### THE EFFECT OF ANIONIC DETERGENTS AND TRYPSIN ON MITOCHONDRIA OF NORMAL AND TUMOUR TISSUES

V. MUTOLO AND F. ABRIGNANI

*From the Laboratory of Biology, Centro Tumori, Palermo, Italy*

Received for publication April 14, 1958

IN previous studies (Mutolo and Abrignani, 1957) it has been observed that mitochondria isolated from 256 Walker tumour cells are more susceptible to the action of trypsin than are those from normal rat liver. It was also found that the decrease of optical density of a mitochondrial suspension in the presence of anionic detergents was more marked with mitochondria derived from normal liver tissue than with mitochondria from tumour cells. This result suggested a structural-chemical difference between the proteins of the membrane of normal and tumour mitochondria.

Recently, however, Cooper and Tapley (1957) have compared the response of the mitochondria from various normal tissues to several swelling agents and found considerable differences.

These observations called for caution in the interpretation of our results and it seemed imperative to check their general validity with an extensive study of the mitochondria isolated from several normal tissues and organs and several strains of tumours. The results of such investigations are reported in the present paper and they seem to lend support to our previous conclusions.

### MATERIALS AND METHODS

*Normal tissues.*—The following tissues were used : liver, spleen, embryonic tissues, and regenerating liver of normal albino rats ; liver, spleen, and kidney of normal mice ; and tissue cultures of human liver cells (strain Chang).

For the study of mitochondria of regenerating liver, hepatectomy was performed on a group of rats and the animals were sacrificed after 10 days.

The medium for cellular growth of the cells of the Chang liver strain was 55 per cent Hank's balanced salt solution, 20 per cent lacto-albumin, 20 per cent inactivated horse serum, and 5 per cent human serum.

*Tumour tissues.*—Mitochondria were prepared from the following tumours : hepatoma ascites tumour, 256 Walker tumour, and transplantable myeloma of rats ; Sarcoma 180, and Ehrlich ascites tumour of mice ; and tissue cultures of malignant human cells (HeLa strain).

For these experiments only the solid tumours free of necrotic tissue were excised. The ascitic fluids of hepatoma and Ehrlich tumour and the suspension of cells cultivated *in vitro* were centrifuged at 700 × g. for 10 minutes and the supernatants were removed ; the cellular sediments were used.

For growth of the cells of the HeLa strain the liquid medium consisted of 199 Morgan's solution containing 20 per cent calf serum, and 2 per cent chicken embryo extract, according to F. G. Oddo (personal communication).

*Preparation of mitochondria.*—The animals were killed by decapitation and the tissues were collected immediately. The tissues and the cellular sediments were homogenized in a glass homogenizer with a teflon plunger. The homogenization was carried out at 0° C. in nine volumes of 0·44 M sucrose in 0·1 M citrate buffer (pH 6·3) containing $10^{-3}$ M versene. Nuclei and cell debris were sedimented at 700 × g. for 10 minutes (in the MSE refrigerated centrifuge) and the supernatant was then centrifuged at 10,000 × g. for 20 minutes. The sedimented mitochondrial pellet was homogenized in five volumes of 0·44 M sucrose in 0·1 M citrate buffer

FIG. 1.—Changes of the optical density ($D_{520}$) of mitochondria suspensions in 0·3 M sucrose. (*a*) Chang cells; (*b*) mouse spleen; (*c*) rat embryo; (*d*) rat spleen; (*e*) mouse kidney; (*f*) mouse liver; (*g*) regenerating rat liver; (*h*) rat liver.

(pH 6·3) and centrifuged at 24,000 × g. for 20 minutes. The final sediment was suspended with a homogenizer in 0·5 M sucrose in 0·02 M tris buffer pH 7·4 and diluted with sucrose so that the optical density ot 520 mμ of the incubation mixture was about 0·250. The mitochondria were always used immediately after preparation.

*Incubation procedure.*—0·1 ml of the final mitochondrial suspension was added to 3·0 of 0·3 M sucrose in tris buffer pH 7·4. After mixing, the optical densities were read at 520 mμ in a Beckman DU Spectrophotometer at 30 seconds, 5, 10, 15 minutes. Then 0·1 ml. of one of the following solutions was added : 0·15 per cent crystalline trypsin (Armour) ; 0·08 per cent duponol in tris buffer pH 7·4 ; 0·27 M sodium deoxicholate in tris buffer ; tris buffer alone (as control). The suspensions were mixed and the optical densities were read every 5 minutes for 15 minutes.

The incubation was performed at room temperature (19–20° C.); for the duration of the experiments no agglutination was observed.

The per cent decrease of the optical density has been calculated minus that observed in a control sample containing only 0·3 M sucrose.

RESULTS

*Normal tissues*

Mitochondria isolated from the organs of normal animals and suspended in 0·3 M sucrose showed considerable variability in their swelling response. As it

FIG. 2.—Changes of the optical density ($D_{520}$) of mitochondria suspensions in 0·3 M sucrose. (*a*) HeLa cells; (*b*) Sarcoma 180; (*c*) Ehrlich ascites; (*d*) hepatoma ascites; (*e*) 256 Walker; (*f*) myeloma.

appears from Fig. 1, at this concentration of sucrose, while the mitochondria of normal and regenerating rat liver and mice liver exhibit a quite pronounced swelling, those of mouse kidney, rat spleen, rat embryo, and mouse spleen undergo very little swelling, and the mitochondria of Chang strain do not seem to undergo any swelling at all.

As it is shown in Fig. 3, both duponol and sodium deoxicholate cause a considerable decrease of the optical density of the mitochondrial suspensions of normal tissues, which is more marked in the case of duponol.

FIG. 3.—Effect of duponol (□□) and sodium deoxicholate (■■) on the optical density of mitochondria suspensions.

FIG. 4.—Effect of trypsin in the optical density of mitochondria suspensions.

On the other hand, the swelling of the mitochondria of normal tissues was in no case increased by trypsin, at least at the concentration used in the present experiments. In some experiments even a protective action was observed (Fig. 4).

*Tumour tissues*

The data summarized in Fig. 2 show that mitochondria isolated from all the above mentioned tumours undergo very little, if any, swelling when suspended in 0·3 M sucrose. When duponol or sodium deoxicholate were added, a slight decrease of the optical density of the suspensions occurred but in all cases such a decrease was less than that observed with the mitochondria of normal cells.

On the contrary, a definite decrease of the optical density upon the addition of trypsin was always observed in the suspensions of tumour mitochondria (Fig. 4).

### CONCLUSIONS

These results first of all confirm those of Cooper and Tapley (1957) in that the swelling response of the mitochondria of normal tissues in 0·3 M sucrose varies, according to the tissue from which they have been prepared. A similar difference is also noted in their response to anionic detergents. This seems to indicate differences in the physiological and chemical properties of the membrane of mitochondria from different tissues.

However, all normal tissues have proved consistently to be entirely refractory to the action of trypsin and this seems to be one of the most peculiar differences between mitochondria from normal and neoplastic tissues.

In fact, the present experiments indicate quite clearly, in confirmation of our previous results, that the mitochondria from all tumour cells studied thus far are quite sensitive to the attack of trypsin. This seems to be a characteristic which distinguishes mitochondria from normal and tumour cells and which may explain, at last partially, the altered metabolic activities of the tumour cells. It also seems of interest to draw attention to the fact that mitochondria of the cells of the HeLa strain behave as those of all other tumour cells, in spite of having been adapted to life *in vitro*.

### SUMMARY

Mitochondria isolated from different normal and tumour tissues have shown a swelling response in 0·3 M sucrose and in presence of anionic detergents according to the tissue from which they have been prepared.

The mitochondria isolated from all tumour cells studied were quite sensitive to the action of trypsin.

We wish to thank to Professor A. Monroy for valuable advice and helpful discussions.

The hepatoma ascites tumour was kindly supplied to us by Dr. F. Zajdela, to whom we wish to express our sincere thanks.

This investigation was supported in part by a grant from the Lega italiana per la lotta contro i tumori.

### REFERENCES

COOPER, C. AND TAPLEY, D. F.—(1957) *Biochim. Biophys. Acta*, **25**, 426.

MUTOLO, V. AND ABRIGNANI, F.—(1957) *Brit. J. Cancer*, **11**, 590.